# Profound Treg perturbations correlate with COVID-19 severity

**DOI:** 10.1073/pnas.2111315118

**Published:** 2021-08-25

**Authors:** Silvia Galván-Peña, Juliette Leon, Kaitavjeet Chowdhary, Daniel A. Michelson, Brinda Vijaykumar, Liang Yang, Angela M. Magnuson, Felicia Chen, Zachary Manickas-Hill, Alicja Piechocka-Trocha, Daniel P. Worrall, Kathryn E. Hall, Musie Ghebremichael, Bruce D. Walker, Jonathan Z. Li, Xu G. Yu, MGH COVID-19 Collection & Processing Team, Diane Mathis, Christophe Benoist

**Affiliations:** ^a^Department of Immunology, Harvard Medical School, Boston, MA 02115;; ^b^Imagine Institute, INSERM UMR 1163, University of Paris, 75015 Paris, France;; ^c^Massachusetts Consortium on Pathogen Readiness, Boston, MA 02115;; ^d^Ragon Institute of Massachusetts General Hospital, Massachusetts Institute of Technology, and Harvard, Cambridge, MA 02139;; ^e^Department of Medicine, Massachusetts General Hospital, Boston, MA 02114;; ^f^HHMI, Center for the AIDS Programme of Research in South Africa, Chevy Chase, MD 20815;; ^g^Brigham and Women’s Hospital, Harvard Medical School, Boston, MA 02115

**Keywords:** COVID-19, Tregs, tumor Tregs, FoxP3

## Abstract

The hallmark of severe COVID-19 is an uncontrolled inflammatory response, resulting from poorly understood immunological dysfunction. We hypothesized that perturbations in FoxP3^+^ T regulatory cells (Treg), key enforcers of immune homeostasis, contribute to COVID-19 pathology. Cytometric and transcriptomic profiling revealed a distinct Treg phenotype in severe COVID-19 patients, with an increase in Treg proportions and intracellular levels of the lineage-defining transcription factor FoxP3, correlating with poor outcomes. These Tregs showed a distinct transcriptional signature, with overexpression of several suppressive effectors, but also proinflammatory molecules like interleukin (IL)-32, and a striking similarity to tumor-infiltrating Tregs that suppress antitumor responses. Most marked during acute severe disease, these traits persisted somewhat in convalescent patients. A screen for candidate agents revealed that IL-6 and IL-18 may individually contribute different facets of these COVID-19–linked perturbations. These results suggest that Tregs may play nefarious roles in COVID-19, by suppressing antiviral T cell responses during the severe phase of the disease, and by a direct proinflammatory role.

## Significance

Regulatory T cells (Tregs) are responsible for restraining excessive inflammation, a hallmark of COVID-19. We identified a striking phenotype in Tregs from patients with severe disease, as well as an interesting role for interleukin (IL)-6 and IL-18. An increased suppressive profile, including increased Treg proportions, combined with the expression of proinflammatory mediators, distinguished severe patients and persisted in some of those recovered. This phenotype is in notable similarity to that found in tumor-infiltrating Tregs, which are generally associated with poor prognosis, and suggests both a detrimental role for these cells in COVID-19 as well as a potential explanation for some of the still largely unexplored complications associated with recovery.

COVID-19 resulting from SARS-CoV2 infection, is a major global health challenge. Many infected individuals remain asymptomatic or present with only mild flu-like symptomatology, appearing 5 to 10 d after exposure, and clearing in over 1 to 2 wk. But this is followed, in patients with more severe disease, by immunopathology and immune dysregulation of poorly understood origin, a major root of the fatality rate of 1 to 12% in different locales. Broad immune-profiling studies (1–3) have documented several immune phenomena that track with disease severity: lymphopenia (4), myeloid cell abnormalities (5), impaired response to interferon (6), and high levels of inflammatory cytokines (“cytokine storm”) (7). Multiomic studies have shown that these manifestations are embedded within multitrait immunotypes (1–3), complicating mechanistic inference and the definition of potential therapies.

Regulatory T cells (Tregs) expressing the transcription factor FoxP3 are essential to maintain immunologic homeostasis, self-tolerance, and to prevent runaway immune responses (8). Tregs regulate the activation of several lineages of the innate and adaptive immune systems through several effector mechanisms (9). Furthermore, particular populations of “tissue Tregs” provide homeostatic regulation in several nonimmunological tissues, controlling inflammation and promoting harmonious tissue repair (10). On the other hand, Tregs can also prove noxious, as evidenced most clearly by their suppression of effective cytotoxic responses in tumors, situations in which they adopt a distinctive phenotype (11–13). They can also have paradoxical effects on antiviral responses (14, 15)

In light of these contrasting influences, we hypothesized that Tregs might contribute to the balance of disease manifestations that distinguish mild from severe outcomes after SARS-CoV2 infection: for example, by insufficiently curtailing the inflammatory component, by overcurtailing the antiviral response, or by phenotypic destabilization. We thus performed a deep immunologic and transcriptional analysis of circulating blood Tregs across a cohort of confirmed COVID-19 patients.

## Results

### More Tregs, More FoxP3 in Severe COVID-19 Patients.

**Fig. 1.**
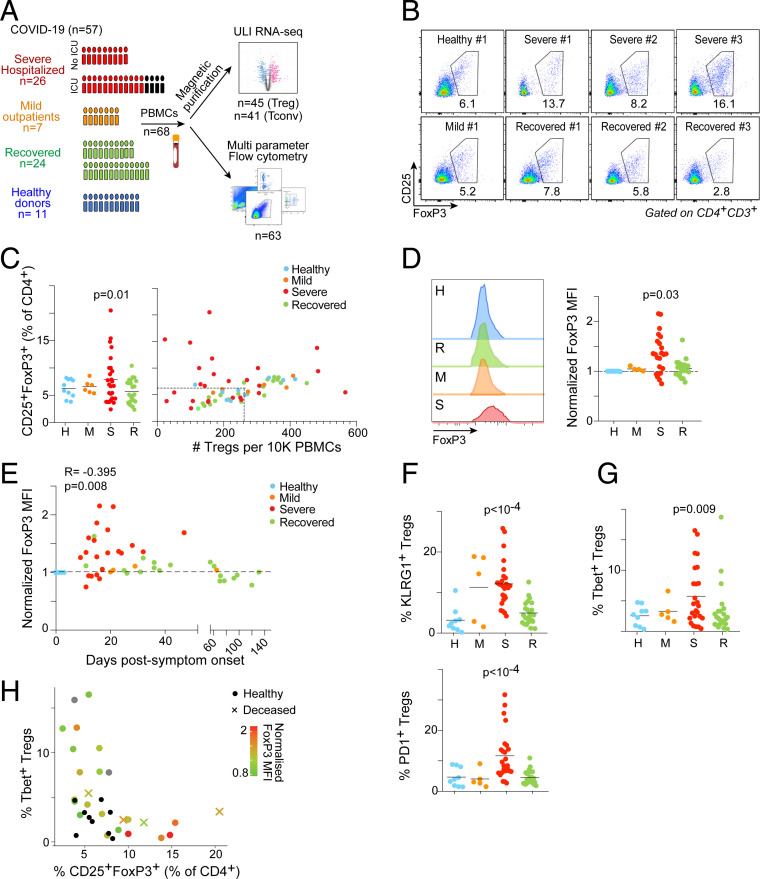
Treg overrepresentation and FoxP3 induction in COVID-19 patients. (*A*) Experimental approach. Tregs from PBMCs across mild, severe, and recovered COVID-19 patients, compared to HD, were assessed by flow cytometry, as well as by RNA-seq. (*B*) Representative flow cytometry plots of CD25^+^FoxP3^+^ Tregs from COVID-19 patients’ PBMCs. (*C*) Proportions (*Left*) and proportions vs. absolute numbers (*Right*) of Tregs as measured by flow cytometry across donors; H, HD; M, mild; R, recovered; S, severe. *P* values from random permutation test quantitating number of outlier values relative to distribution in HDs. (*D*) FoxP3 expression, measured as MFI in CD127^lo^CD25^+^ Tregs. Representative flow cytometry profiles (*Left*) and quantification (*Right*); Mann–Whitney *P* values. (*E*) Correlation between FoxP3 expression and days post disease symptoms onset across COVID-19 patients; Pearson correlation test (*F*) Proportion of KLRG1^+^ (*Upper*) and PD1^+^ (*Lower*) Tregs as determined by flow cytometry across COVID-19 patients; significance computed as for *C*. (*G*) Proportion of Tbet^+^ Tregs as determined by flow cytometry across COVID-19 patients; significance computed as for *C*. (*H*) Correlation between percentage of CD25^+^FoxP3^+^ Tregs (*x* axis), percentage of Tbet^+^ Tregs (*y* axis), and FoxP3 expression as MFI (color gradient) within severe COVID-19 patients. Healthy controls depicted in black dots and patients with fatal outcome by an “x”; those with an unavailable normalized FoxP3 MFI measurement are depicted as gray dots.

We performed a deep immunologic and transcriptional analysis of circulating blood Tregs across a cohort of confirmed COVID-19 patients (*n* = 57) (Fig. 1*A* and Dataset S1): mild, outpatients; severe, hospitalized, 65% of which in intensive care (ICU), mostly sampled during the cytokine storm period; and recovered, virus-negative convalescents. Flow cytometry, with a multiparameter panel that parsed CD25^+^FoxP3^+^ Tregs and their different phenotypes (gating strategies in *SI Appendix*, Fig. S1*A*), revealed several perturbations (representative plots in Fig. 1*B*). First, several severe patients showed increased Treg proportions among CD4^+^ T cells: for some as an increased proportion only (likely resulting from preferential resistance among CD4^+^ T cells during lymphopenia); for others, from true numerical increase relative to healthy donors (HD) (Fig. 1*C*). This conclusion differs from a recent analysis of CD4^+^ T cells that up-regulated CD69/4.1BB after 24-h culture with a multipeptide mixture, hence very different from the present ex vivo study and possibly complicated by selection in culture (16). Recovered patients largely reverted to baseline.

Second, expression of both FoxP3 and CD25 varied in severe patients, bidirectionally for CD25 (*SI Appendix*, Fig. S1*B*), but as a reproducible increase of FoxP3 mean fluorescence intensity (MFI) (Fig. 1*D*). Increased FoxP3 expression coincided with the increase in Treg percentage in most but not all patients (*SI Appendix*, Fig. S1*C*), and was observed within a broad window of 30 to 50 d after symptom onset, including some recently recovered patients (Fig. 1*E*). It was not related to body/mass index (BMI), a risk factor for COVID-19 (*SI Appendix*, Fig. S1*D*), but coincided with disease severity, particularly marked in ICU-admitted patients (*SI Appendix*, Fig. S1*E*), and in lymphopenic cases (*SI Appendix*, Fig. S1*F*). No dependence of these phenotypes was found on any particular treatment, in particular glucocorticoids (Dataset S1). FoxP3 expression was not directly correlated with blood levels of the inflammatory marker C-reactive protein (CRP), but essentially all FoxP3^hi^ patients had elevated CRP (*SI Appendix*, Fig. S1*G*). Therefore, severe COVID-19 entails a striking induction of FoxP3 expression in Tregs.

We examined the expression of several markers and transcription factors (TF) to further assess Treg evolution during COVID-19. CD45RA, which marks naïve Tregs, was reduced in patients, especially in those with heightened Tregs and FoxP3 (*SI Appendix*, Fig. S1 *H* and *I*). Expression of the activation markers KLRG1 and PD1 was increased (Fig. 1*F*), although not necessarily coordinately (*SI Appendix*, Fig. S1*J*). Suppression of specific T cell effector functions is associated with the expression in Tregs of the same key TFs that drive those conventional T cell (Tconv) functions (8). No patient sample showed significant expression of Bcl6, which marks T follicular regulators (17, 18). But Tbet, expressed in Tregs that preferentially control Th1 responses was overrepresented in severe patient Tregs (Fig. 1*G*). Interestingly, severe patients with frequent Tbet^+^ Tregs were distinguished from those with highest Treg overrepresentation and FoxP3 overexpression (Fig. 1*H*). Ultimately, deceased patients were mostly found in the latter group. Thus, severe COVID-19 seems to elicit divergent deviations among Treg cells. We surmise that the presence of Tbet^+^ Tregs is related to the control of Th1 antiviral response among effector cells.

### Accentuated Treg Transcriptome in Severe COVID-19 Patients.

**Fig. 2.**
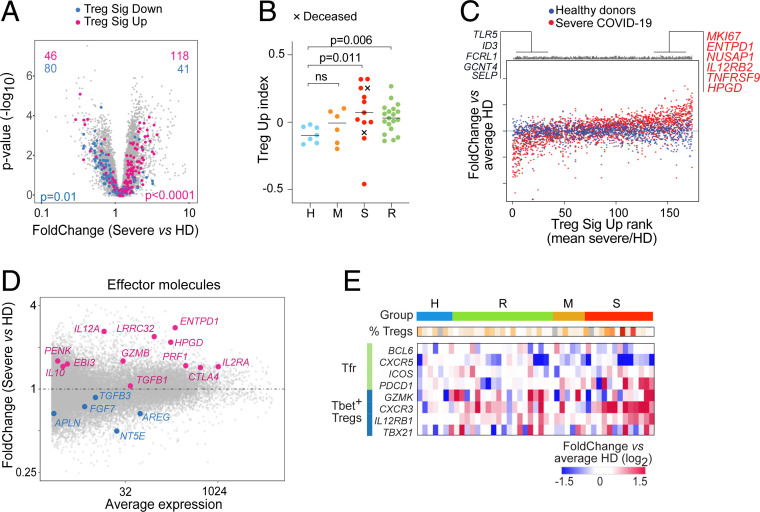
A “super-Treg” identity in Tregs from severe COVID-19 patients. Tregs from COVID-19 patients and HD were magnetically purified for population RNA-seq. (*A*) Fold-change vs. *P* value (volcano) plot of gene expression in Tregs from severe COVID-19 patients compared to HD. Genes from Treg-Up signature (red) and Treg-Down signature (blue) are highlighted (54); *P* values from Fisher’s exact. (*B*) Treg-Up index was computed by averaged normalized expression of all signature genes in Tregs from COVID-19 patients and HD. Patients with fatal outcomes depicted as an “x”; *P* values from Mann–Whitney test. (*C*) Ranked expression of Treg-Up signature genes in Tregs from severe COVID-19 patients (red) and HD. The *y* axis corresponds to the expression in each donor Treg relative to the mean of HD Tregs. Genes are ranked by their average ratio in severe COVID-19 patients. Each dot is one gene in one donor Treg sample. (*D*) Fold-change vs. average expression plot from severe COVID-19 patient Tregs compared to HD. Up-regulated (red) and down-regulated (blue) Treg effector molecule transcripts highlighted. (*E*) Heatmap of the expression of transcripts typical of T follicular regulators (Tfr) and Tbet^+^ Treg in Tregs from each donor (as fold-change vs. mean expression in HD). One column per donor, with severity groups color-coded and percentage of FoxP3^+^CD25^+^ Tregs from the flow cytometry indicated.

To decipher the functional consequences of FoxP3 overexpression in Tregs from severe COVID-19 patients, we generated 86 RNA-sequencing (RNA-seq) transcriptome profiles, passing quality thresholds, of blood Treg (CD4^+^CD25^hi^CD127^lo^) or Tconv (CD4^+^CD25^−^). Donors largely coincided with those analyzed above, and we opted to profile purified populations rather than single-cell (higher throughput, lower processing/computational costs) after magnetic purification (*SI Appendix*, Fig. S2*A*). The datasets matched profiles from flow-purified Tregs, with the usual differential expression of “Treg signature” genes (*SI Appendix*, Fig. S2*B*). In line with the cytometry, Tregs from severe COVID-19 patients showed higher *FOXP3* expression (*SI Appendix*, Fig. S2*C*). This overexpression had a consequence: Tregs from severe COVID-19 patients displayed a heightened expression of Treg signature transcripts (Fig. 2*A*), reflected by a high TregSignature index, most markedly biased in the severe group, but also in mild and recovered patients (Fig. 2*B*), and correlating with *FOXP3* expression (*SI Appendix*, Fig. S2*C*). This bias was not homogeneous across all Treg-up signature genes, a ranked plot of differential expression in each donor revealing a small subset of TregUp signature genes actually down-regulated in severe COVID-19 patients (including *TLR5*, *ID3*, and *FCRL1*) (Fig. 2*C*). At the other end of the spectrum, several Treg effector or activation transcripts were up-regulated in severe patients (*ENTPD1*, *HPGD*, *IL12RB2*). Most transcripts encoding known Treg effector molecules were up-regulated, albeit to various degrees (Fig. 2*D*), with the marked exception of *AREG*, a dominant player in Treg promotion of tissue repair (19, 20).

We also examined transcripts associated with effectiveness at suppressing different Tconv functions (8). Transcripts associated with T follicular regulators were largely unaffected in Tregs from severe patients (Fig. 2*E*), with the exception of *PDCD1* (encodes PD1), consistent with cytometry results. In contrast, they generally up-regulated markers related to Th1 suppression (*CXCR3*, *GZMK*, *IL12RB1*, or *TBX21* [encodes Tbet]), especially marked in patients with low percentage of Tregs (Fig. 2*E*), in line with the overrepresentation of Tbet^+^ Tregs noted above. Profiles from Tconv cells did not denote a particular bias toward any Th phenotype (*SI Appendix*, Fig. S2 *D* and *E*). Thus, Treg traits observed in the flow cytometry data were confirmed by the transcriptomic signature of these Tregs, which tends toward a supersuppressive phenotype in severe COVID-19 patients.

### A Distinct COVID-19 Disease Treg Signature Correlates with Severity.

**Fig. 3.**
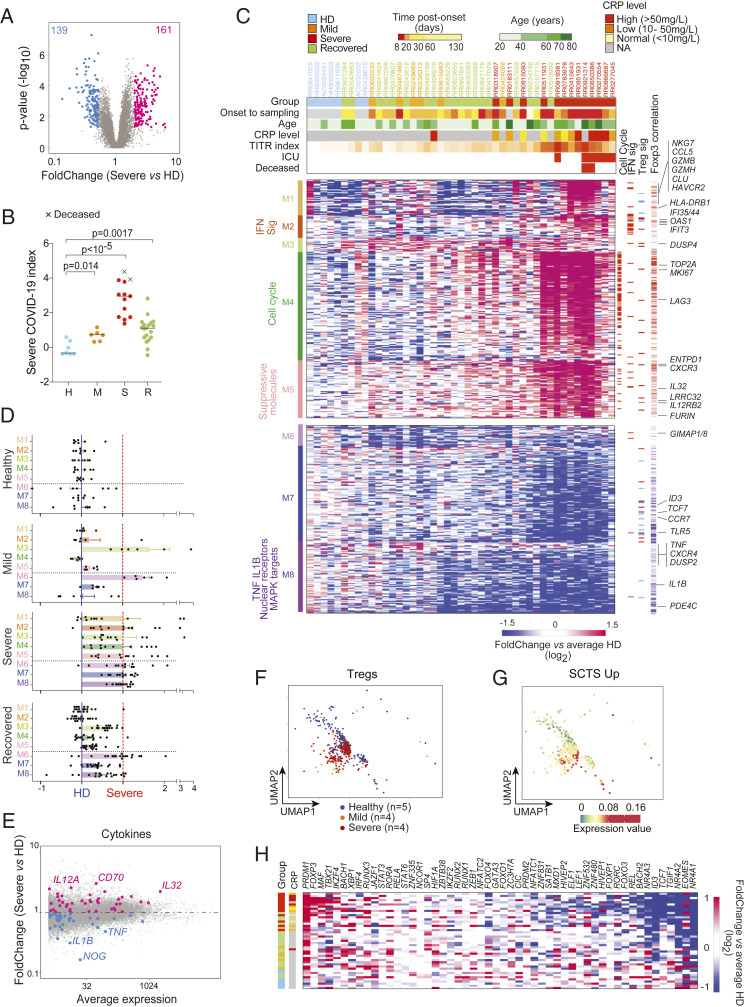
Broad perturbations of Treg transcriptomes in severe COVID-19. (*A*) Fold-change (FC) vs. *P* value plot of gene expression in Tregs from severe COVID-19 patients compared to HD. Differentially expressed genes are highlighted (at an arbitrary threshold of *P* < 0.05, FC > 2, or < 0.5). (*B*) Severe COVID-19 index (computed from relative expression of selected genes from *A*) in Tregs from COVID-19 patients and HD; *P* values from Mann–Whitney test. (*C*) Clustered heatmap of differentially expressed genes (selected as *P* < 0.05, FC > 2 or < 0.5) across all donors (as ratio to mean of HD values). Each column represents one donor. Top ribbons indicate for each individual: severity group, days from symptom onset to sample collection, age, CRP level at sampling, tumor infiltrating Treg index, ICU admission, and final outcome (deceased in red). Left ribbon indicates the coregulated modules and their dominant composition; right ribbons denote transcripts related to cell cycle, interferon responsive genes, Treg signature genes, and Pearson correlation of each gene’s expression to FOXP3 MFI across all samples. (*D*) Representation of the average expression of each module across each group (from *C*, mean and SEM). Score computed independently for each module, where 0 corresponds to the average expression of the module in HD Tregs and 1 the average expression in severe COVID-19 Tregs (red line). (*E*) Changes in expression of cytokine-encoding transcripts (on a fold-change vs. average expression plot, severe COVID-19 vs. HD Tregs). (*F*) Treg cells extracted from single-cell RNA-seq dataset (GSE150728) displayed as a two-dimensional UMAP. The samples are color-coded by group. (*G*) Same plot as *F*, where each cell is color-coded according to expression of the SCTS-Up signature genes. (*H*) Expression of selected transcription factors in Tregs from each donor (as ratio to mean of HD values). Each column corresponds to one individual, with severity group color-coded and a ribbon indicating CRP levels.

We then asked more generally what changes, beyond Treg signature and effector transcripts, characterized Tregs in severe COVID-19 patients, relative to HD (Fig. 3*A* and Dataset S2) (hereafter “Severe COVID19 Treg Signature,” SCTS); only a minority of these transcripts belong to the classic Treg signature analyzed above. An SCTS index computed from this gene set was high in all severe patients, but also persisted in muted fashion in many recovered patients (Fig. 3*B*), correlating with *FOXP3* expression (*SI Appendix*, Fig. S3*A*). The index was not directly related to patient age or BMI (*SI Appendix*, Fig. S3*B*). Grouping SCTS transcripts into a biclustered heatmap (Fig. 3*C*) revealed several interesting features. Most donors clustered according to severity group, in relation to disease duration (more marked deviation at shorter times), but not to donor age (except inasmuch as severe patients were generally older). Transcripts could be parsed into eight different modules of distinct composition. Among these, module M4 was almost exclusively composed of cell cycle-related transcripts, and strongly correlated with FoxP3 MFI, indicating that Tregs in COVID-19 patients are highly proliferative, plausibly a compensation for the lymphopenia in many severe patients. M2 mostly included interferon-stimulated genes (ISGs). The other coregulated modules included transcripts related to immune cell cross-talk (Fig. 3*C*, *Right*). Expression of these modules evolved differently during the course of the disease (Fig. 3*D*). The ISGs in M2 were mostly overrepresented at early times, consistent with sharp induction at the early phase of antiviral responses. Changes in other modules, especially M3 and M6, were shared across all disease stages, indicating a broader and longer-lasting perturbation of the Treg pool, likely caused by secondary consequences of the disease rather than by the virus itself. To assess how the imprint evolves over the course of disease in the same patient, we performed RNA-seq of sequential samples from a few initially severe patients. The SCTS index was higher at the early stage of ICU hospitalization and generally decreased with time (*SI Appendix*, Fig. S3*C*).

Focusing on cytokines produced by Tregs, *IL10* was only modestly induced, but *CD70* (CD27L) and *IL32* dominated (Fig. 3*E*). The latter was intriguing in this context of the cytokine storm that occurs in these severe patients, since it is mainly a proinflammatory mediator, in positive feedback with interleukin (IL)-6 and IL-1β (21). To answer the common question (whether transcriptional changes reflect a shift in subset balance or are all shared by all Tregs), we reanalyzed a single-cell RNA-seq peripheral blood mononuclear cell (PBMC) dataset from COVID-19 patients (22), drilling down on identifiable Treg cells (*SI Appendix*, Fig. S3*D*). Tregs from severe patients were generally shifted in the UMAP projection relative to HD (Fig. 3*F*) and displayed an up-regulation of the SCTS (Fig. 3*G* and *SI Appendix*, Fig. S3*E*). *IL32* was again one of the dominant up-regulated transcripts (*SI Appendix*, Fig. S3*F*).

### COVID-19 Tregs as Tumor Tregs.

We then attempted to better understand the origin of the SCTS. Comparing changes in Treg and Tconv cells showed some induction in the latter (particularly for the inflammation-related components of M1), but overall much less than in Tregs (*SI Appendix*, Fig. S3*G*), indicating that the SCTS is largely Treg-specific. With regard to the cytokines, the up-regulation of *IL10* and *IL32* were specific to Tregs, as was the down-regulation of inflammatory cytokines (*TNF*, *IL1B*) (*SI Appendix*, Fig. S2*E*). Interestingly, the SCTS was associated with changes in several TFs previously associated with differential gene expression in activated Tregs (Fig. 3*H*). *PRDM1* (also known as BLIMP1) and *MAF* were up-regulated, while *ID3*, *TCF7*, and *BACH2* were repressed, consistent with previous reports (23–27). More unexpected was the strong down-regulation of *NR4A1*, normally an indicator of T cell receptor (TCR) signaling, which may indicate a decoupling of TCR-delivered signals.

**Fig. 4.**
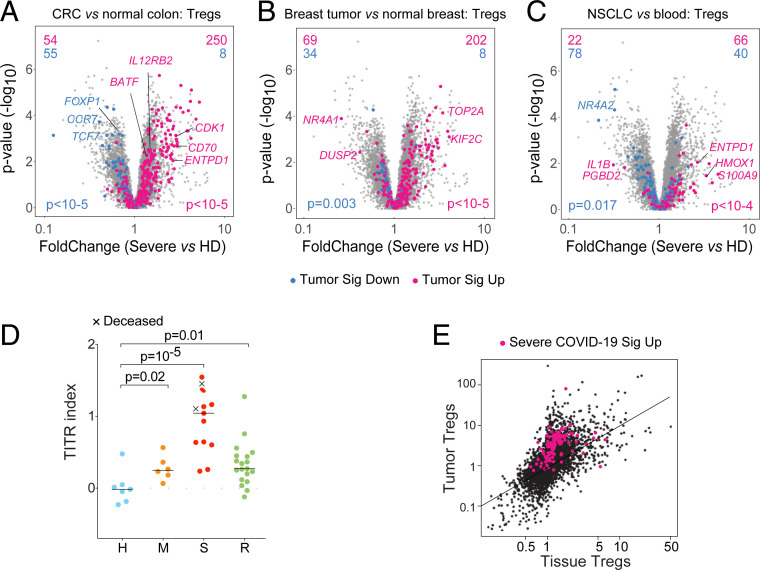
The severe COVID-19 Treg transcriptome overlaps with that of TITR. (*A–C*) Volcano plots comparing Tregs from severe COVID-19 patients relative to HD, highlighted with signature genes from: (*A*) colorectal cancer (CRC) vs. colon Tregs (GSE116347); (*B*) breast tumors vs. normal breast Tregs (GSE89225); (*C*) nonsmall-cell lung cancer (NSLC) vs. blood Tregs (PRJEB11844); *P* values from Fisher’s exact. (*D*) TITR index in Tregs from COVID-19 patients and HD; *P* values from Mann–Whitney test. (*E*) Highlight of the severe SCTS Up signature (red) on a plot comparing transcriptome shifts in TITRs vs. tissue-resident Tregs (see ref. 13).

Gene enrichment analyses using a curated database of transcriptome variation in CD4^+^ T cells brought forth 229 datasets with significant overlap to the SCTS (*SI Appendix*, Fig. S4*A*). Besides the expected interferon-related gene sets related to M2, many were related to Tconv and Treg activation, mostly overlapping with M4 and M5. Most intriguing were overlaps with datasets from tumor-infiltrating Tregs (TITR). Indeed, the expression of a signature that distinguishes colorectal TITR from normal colon tissue (13) was strikingly biased in Tregs from severe COVID-19 patients (Fig. 4*A*). The same bias was found with gene sets that distinguish breast and lung TITRs from blood Tregs (11, 12) (Fig. 4 *B* and *C*). A hypoxia-induced gene set, a hallmark of tumors, was also enriched in Tregs from severe COVID-19 patients (*SI Appendix*, Fig. S4*B*). Computing a TITR index from the colorectal tumor gene set showed that biased expression of tumor Treg transcripts was present in all severe patients, particularly in those eventually deceased (Fig. 4*D*). This index remained slightly perturbed after recovery, and was highly correlated with the SCTS (*SI Appendix*, Fig. S4*C*). Conversely, with the exception of M1 and M6, all SCTS modules showed biased expression in tumor Tregs relative to normal tissue (*SI Appendix*, Fig. S4*D*), indicating widespread sharing not solely limited to activation-related transcripts.

TITRs and tissue Tregs are transcriptionally similar, suggesting the possibility that the SCTS simply denoted Tregs circulating en masse from the lung tissue. This was not the case, however; when highlighted in a direct comparison of tissue and tumor Tregs (from ref. 13), the SCTC was clearly biased toward the tumor angle (Fig. 4*E*). Furthermore, a set of transcripts shared by tissue Tregs showed little enrichment in Tregs from severe COVID-19 patients (*SI Appendix*, Fig. S4*E*). This bidirectional matching shows that the COVID-19 disease appears to be turning blood Tregs into some equivalent of tumor Tregs.

### IL-6 and IL-18 as Inducers of the Treg COVID-19 Phenotype.

We next set out to identify the signals that might be responsible for these Treg perturbations. Given the similarities between TITRs and Tregs from severe COVID-19 patients, we centered the search on factors that Tregs might encounter in both tumors and SARS-CoV2 infection. Hypoxia was one such factor, as it is a hallmark of tumors and an important factor in severe COVID-19 (28), and can promote Treg suppressive function (29). Correspondingly, high levels of lactic acid are present in tumors, where they have been reported to affect Tregs (30), and in COVID-19 patients (31). Several inflammatory cytokines and chemokines are also present at elevated levels in both tumors and blood from COVID-19 patients (32–36).

**Fig. 5.**
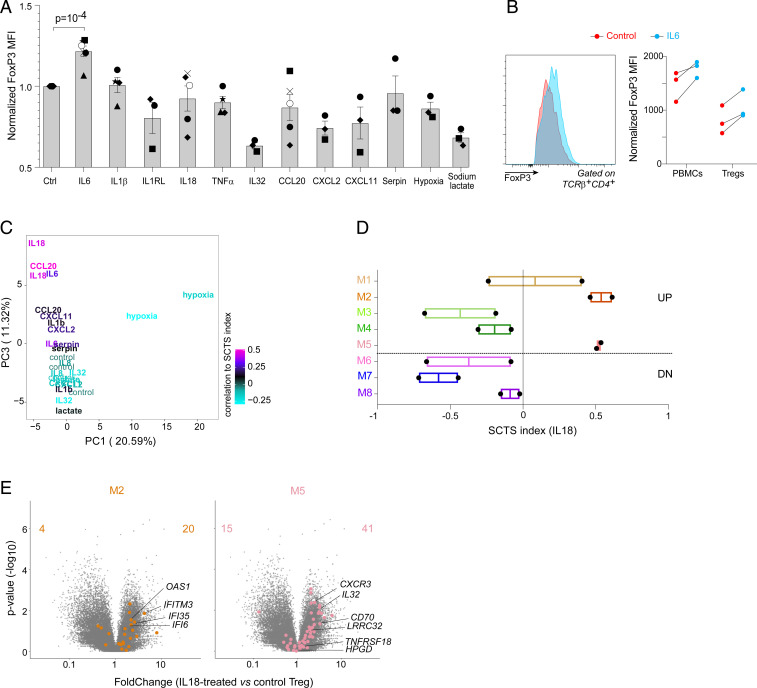
IL-6 and IL-18 partially recapitulate the severe COVID-19 Treg phenotype in vitro. (*A*) PBMCs from HDs were left untreated (ctrl) or treated for 24 h with a series of candidate cytokines or metabolites, or cultured in a hypoxic chamber. FoxP3 expression was assessed in gated Tregs by flow cytometry (MFI shown). Different donors depicted by different symbols. (*B*) Whole PBMCs or purified CD127^lo^ CD25^+^ Tregs were cultured with IL-6 as and analyzed as in *A*. Representative profiles from FoxP3^+^ CD25^+^CD4^+^ cells from control or IL-6–supplemented cultures. (*C*) RNA-seq was performed on Tregs cultured for 24 h with several cytokines or in hypoxic conditions. The effects were parsed by PCA of normalized transcript counts, and the scores used for plotting, color-coded to indicate the SCTS index of each sample. (*D*) Average score of each SCTS module among the IL-18–treated Tregs (each dot is one of two replicates). (*E*) Volcano plot comparing IL-18–treated to control, highlighted with genes from SCTS modules 2 and 5.

To test these candidates, we cultured PBMCs from HDs for 24 h with each of the short-listed mediators and conditions, assessing the effects on FoxP3 levels by flow cytometry, or as a deviation of SCTS transcripts in RNA-seq. A few led to Treg cell loss (IL-32, Nicotinamide) (*SI Appendix*, Fig. S5), some decreased FOXP3 expression (IL32, CXCL22) (Fig. 5*A* and *SI Appendix*, Fig. S5), but IL-6 stood out in eliciting a modest but reproducible increase in FoxP3 MFI (Fig. 5*A* and *SI Appendix*, Fig. S5) that reproduced the situation in severe patients in Fig. 1*D*. Titrating IL-6, or adding it in combination with other cytokines, did not enhance this effect. To ask if IL-6 affected Tregs directly—or indirectly by stimulating, for example, myeloid cells in these cultures—we repeated the experiment with purified CD25^+^CD127^lo^ Tregs. The same increase in FoxP3 fluorescence intensity was observed, demonstrating a direct effect of IL-6 on Treg cells (Fig. 5*B*).

RNA-seq was then performed on Treg cells purified from two independent sets of cultures. We applied a principal component analysis (PCA) to identify the axes of variance and identify those that correlate with the SCTS. Tregs subjected to hypoxia showed the most divergence from control, essentially accounting alone for the first component of variance (Fig. 5*C*). Changes induced by hypoxia did not, however, corelate with the SCTS. On the other hand, the SCTS index correlated with the third PC, to which IL-18–treated Tregs contributed the most, closely followed by IL-6 and CCL20 (Fig. 5*C*). IL-18–treated Tregs also had the strongest correlation to the SCTS, (color-coded in Fig. 5*C*), while an anticorrelation was seen for IL-32. Further parsing of the IL-18 effect on individual modules of Fig. 3*D* revealed an induction of two up-regulated modules (M5 and M2, the effector and ISG modules, respectively) (Fig. 5 *D* and *E*). Conversely, the modules down-regulated in COVID patients (M6 and M7, principally) were also repressed by IL-18 (Fig. 5*D*). These results suggest that the Treg perturbations of COVID-19 patients are effected in a complementary manner by several mediators with partial effects: these include IL-6’s up-regulation of FoxP3, and IL-18 inducing the broader signature.

## Discussion

Treg cells in severe COVD-19 patients present a striking Treg phenotype, which associates an up-regulation of FoxP3 expression with a distinctive transcriptional signature that bears much similarity to tumor Tregs. Two flow cytometric studies also recently noted increased Treg proportions and activation status correlating with COVID-19 severity (37, 38). The overall transcriptional signature we observe is a broad one, with an induction of ISGs (not unexpected in a context of viral infection), but also of cell proliferation, and of heightened effector functions (*ENTPD1*, *LAG3*, *LRRC32*). Several tumor necrosis factor (TNF) receptor family members, which have important roles in Treg function and homeostasis, are among the induced SCTS transcripts. Also, consistently up-regulated is *CXCR3*, the receptor for CXCL10, one of the soluble mediators most induced in severe COVID-19 blood (39).

These observations raise two key questions. First, how do the perturbations arise? They are not due to viral infection of the Tregs (no viral RNA reads to speak of in these datasets). They seem unconnected to therapy, as none of the therapies administered to these patients correlate with the Treg traits. More likely, the phenotype is induced by the immunologic milieu in these patients, and this uniquely in Tregs since Tconvs are far less branded. TCR-mediated stimulation seems unlikely, given the widespread effect on Tregs in the single-cell data, which likely transcends clonotypic specificity, and the strong loss of Nur77 (*NR4A1*). Our results suggest that a combination of factors is at play, foremost IL-6 and IL-18 (but other factors may contribute as well), each contributing a particular aspect of the perturbed Treg phenotype. The impact of IL-6 in this context is paradoxical at first, as it is generally deemed a Treg antagonist, in particular because it blocks FoxP3 induction by TGF-β/IL-2 in culture (40). More recent studies have painted a more nuanced picture of IL-6 in relation to Treg cells: it is required for the differentiation of the RORγ^+^ Treg subpopulation (41, 42) and can increase their suppressive capabilities (43). Transgenic mice with constitutively high-serum IL-6 have slightly increased levels of Treg cells with good suppressive function (44). A recent study described Treg perturbations in rheumatoid arthritis joints (45) that are reminiscent of those described here (in particular with elevated FoxP3 MFI), an interesting parallel since the arthritic joint is high in IL-6. It would have been valuable to assess Tregs from the lungs of COVID-19 patients directly, but such samples were not available to us.

IL-18 has been shown to promote Treg reparative function via amphiregulin (20), and IL-18 signaling from epithelial cells to Tregs is required for protection against colitis in the RAG transfer model (46). It was recently proposed that IL-18 signaling is an intermediate of the control of proreparative functions in Tregs by Notch4 (47). The IL-18 receptor is preferentially expressed on a subfraction of aTregs, which have preferential thymus-homing capability (48). Our results suggest a broader impact of IL-18 on Treg cells, not only on proreparative pathways, but involving the wider segment of Treg effector functions represented in module M5 (typical Treg transcripts, such as TNFRSF18 or LRRC32). In addition, circulating Tregs from severe COVID-19 patients actually show reduced amphiregulin expression (Fig. 2*D*), indicating that some aspects of IL-18 effects may be counteracted by other components of the COVID-19 cytokine storm.

Second, do these aberrant Tregs contribute to COVID-19 physiopathology? Patients with fewer Tregs, lower FoxP3, and less intense SCTS do better, raising the usual issue of inferring causality from correlation. On the one hand, these Tregs might be beneficial, controlling a cytokine storm that would have been worse without their unusual contribution. Unfortunately, inadequate cell numbers prevented the direct assessment of their suppressive capabilities. On the other hand, their overexpression of FoxP3 and of Treg effector molecules, and their similarity with dominantly suppressive tumor Tregs, suggest that COVID-19 Tregs may overly dampen the antiviral response during the cytokine storm phase (all samples from severe patients profiled were collected in that period), contributing to the secondary re-expansion of disease. Lending credence to this hypothesis, a parallel study of CD8^+^ T cells from the same patients uncovered a dearth of SARS-CoV2 reactive cells in the blood during the acute period (49). There are precedents for rogue Tregs, which acquire proinflammatory characteristics (50), if in opposite conditions of FoxP3 attrition.

In summary, adding a key element to the multifaceted COVID-19 immune response, we identify a unique Treg deviation in COVID-19 patients, which results from the effects of several components of the proinflammatory storm and might impact on COVID-19 pathogeny as it does in tumors.

## Materials and Methods

### Patients Samples and Clinical Data Collection.

Peripheral blood samples from 57 adults infected with SARS-CoV-2 prospectively collected in Massachusetts General Hospital (MGH) were included in this study (Dataset S1). Samples from 11 adult HD were also collected. COVID-19 patients were split into three different groups, depending on the disease severity or the time-course postinfection. All participants provided written informed consent in accordance with protocols approved by the Partners Institutional Review Board and the MGH Human Subjects Institutional Review Board. De-identified Treg analysis was performed per approved Human Subjects Institutional Review Board protocol 15-0504-04.

### PBMC Isolation.

Five to 10 mL of whole blood was collected in K2 EDTA tubes and processed within a few hours. An equal volume of buffer (2 mM EDTA in PBS) and blood was mixed and carefully layered over 5 mL Ficoll Hypaque solution (GE Healthcare). After centrifugation for 20 min at 900 × *g* (with no break) at 25 °C, the mononuclear cell layer was washed twice with excess buffer (three times the volume of the mononuclear cells layer), and centrifuged for 5 min at 400 × *g*. To remove platelets, the cell suspension was then layered over 3 mL FBS, centrifuged for 10 min at 300 × *g*. The pellet was resuspended in 90% FBS-10% DMSO, 5 million cells/mL, and cells stored in liquid nitrogen.

### Treg and Tconv Magnetic Isolation.

Frozen PBMCs samples were processed in batches of 4 to 10 samples, with at least one HD by batch, with strict attention to processing time to avoid cell aggregation, which was otherwise pervasive with samples from severe COVID-19 patients. Five to 10 × 10^5^ PBMCs were resuspended into PBS with 2% FBS and 1 mM EDTA. CD4^+^CD25^hi^CD127^low^ (Treg) were isolated by positive and negative magnetic selection, CD4^+^CD25^-^ (Tconv) by negative selection only. EasySep Human CD4^+^CD127^low^CD25^+^ Regulatory T Cell Isolation Kit (StemCell, #18063) was used following the manufacturer’s instructions. Ten percent of the final isolated fraction was stained with anti-CD4, anti-CD8, anti-CD14, anti-CD19, and live/dead for 15 min at 4 °C, fixed in 1% PFA for 10 min at room temperature, and analyzed by flow cytometry to determine purity and yield (antibodies references below). The remaining 90% of the samples were resuspended in lysis buffer (TCL Buffer [Qiagen] supplemented with 1% 2-Mercaptoethanol) at an average concentration of 500 to 1,500 cells per 5 µL, and stored in a low-binding tube at −80 °C.

### Cell Culture.

PBMCs or flow-isolated Tregs were cultured in DMEM (Thermo Fisher, cat# A1443001) supplemented with 5% dialyzed FBS (Thermo Fisher, cat# A3382001), 1 mM glucose (Sigma), 0.2 mM l-glutamine (GeminiBio) and 10 ng/mL IL7 (BioLegend, cat# 581904). Cells were treated with various mediators for 24 h. More details can be found in *SI Appendix*, *Supplementary Materials and Methods*.

### Flow Cytometry.

Cells were first incubated in 100 μL of PBS with 2 mM EDTA for 15 min with 5 µL Fc Block (Human TruStain FcX, Biolegend, cat #422301) and a 1:500 dilution of Zombie UV viability dye (Biolegend, cat# 423107). They were then washed with FACS buffer (phenol red-free DMEM, 2% FBS, 2 mM EDTA, 10 mM Hepes) and stained at 4 °C for 25 min. After cell surface staining, cells were fixed overnight at 4 °C using 100 μL of Fix/Perm buffer (eBioscience), followed by permeabilization using 1× permeabilization buffer (eBioscience) for 40 min at room temperature in the presence of intracellular antibodies. Antibody details can be found in *SI Appendix*, *Supplementary Materials and Methods*. Data were recorded on a FACSymphony flow cytometer (BD Biosciences) and analyzed using FlowJo 10 software.

### RNA-Seq.

For ex vivo blood Tregs, RNA-seq was performed in two different batches, including samples coming from two to seven different experimental batches (different experiment dates in Dataset S1). After magnetic isolation and using the postisolation flow cytometry data, samples with purity > 65% and expected number of cells >800 were selected for RNA-seq. For cultured Tregs after 24 h of treatment, cells were sorted as DAPI– CD4^+^CD25^hi^CD127^lo^ on a Moflo Astrios Cell Sorter (Beckman Coulter). One thousand cells were double-sorted directly into 5 µL of lysis buffer (TCL Buffer [Qiagen] supplemented with 1% 2-Mercaptoethanol). RNA-seq was performed with 5 µL of the previously described lysate following the standard ImmGen low-input protocol (https://www.immgen.org). Further details related to data analysis can be found in *SI Appendix*, *Supplementary Materials and Methods*.

### Single-Cell RNA-Seq Reanalysis.

Data deposited at data repository cellxgene (51) were used, with the cell annotation provided by Wilk et al. (22), CD4^+^ and CD8^+^ cell clusters were extracted from the processed single-cell data. Using the Seurat pipeline (52), PCs and UMAP coordinates were recomputed for just the CD4^+^ and CD8^+^ T cell populations. From there, *K*-means clustering was recomputed using the FindClusters function with default parameters and the cluster with the highest average expression for a list of core Treg signature genes [*CAPG*, *FOXP3*, *TNFRSF4*, *IFI27L2A*, *TNFRSF18*, *FOLR4*, *TNFRSF9*, *S100A6*, *APOBEC3*, *IKZF2*, *H2AFZ*, *CTLA4*, *LY6A*, *HOPX*, *SERINC3*, and *IL2RA* (53)] was flagged for further analysis. From this Treg cluster, cell averages were calculated across all genes of the Severe COVID-19 (SCTS) up signature, and color-coded on the Treg UMAP space for Fig. 3*H*.

### Statistics.

Unless specified otherwise, data are presented as mean ± SD and tests of associations for different variables between COVID-19 patients and HD were computed 1) using the nonparametric Mann–Whitney test or 2) using randomization test: random values were generated [nrorm() in R] from the mean and SD of log-transformed values in HD controls, testing the frequency of draws that led to a number of observations > 95th quantile of HD values that was equal or greater to the number of such observations in each patient group. Correlation coefficients were from Pearson correlation. Significance of signature overlaps into our dataset was assessed by Fisher’s exact test when computing one signature at a time, or by a hypergeometric test with Benjamini–Hochberg correction when using the large curated CD4^+^ T cell signatures database. Analyses and plots were done using RStudio (v1.2.5019) and GraphPad Prism (v8.4.3), heatmaps generated with Morpheus (https://software.broadinstitute.org/morpheus).

## Supplementary Material

Supplementary File

Supplementary File

Supplementary File

Supplementary File

## Data Availability

The data reported in this paper have been deposited in the Gene Expression Omnibus (GEO) database (accession no. GSE179448).
