# Supplementary material for: Profound Treg perturbations correlate with COVID-19 severity
Source: Proc Natl Acad Sci U S A. 2021 Aug 25;118(37):e2111315118. doi: 10.1073/pnas.2111315118 (PMC8449354; doi:10.1073/pnas.2111315118)
Supplement: Supplementary File [file pnas.2111315118.sapp.pdf]

SUPPLEMENTARY MATERIALS AND METHODS

Patient samples and clinical data collection

SARS-CoV-2 infection was defined by the concomitant presence of a concordant symptomatology and a positive SARS-CoV-2 real-time reverse-transcriptase–polymerase-chain-reaction (RT-PCR) using nasopharyngeal swabs. Healthy donors were dated before December 2019 or defined as no prior diagnosis of or recent symptoms consistent with COVID-19 and a negative PCR test within 3 days pre-collection. Patients were categorized as “severe” if admitted to the hospital for moderate to severe COVID-19 symptoms, either in floor or in intensive care unit, as “mild” group if they were outpatients, as “recovered” if they presented a prior positive COVID-19 PCR test, but the most current PCR test was negative. For inpatients, clinical data, including oxygenation status, medications and the outcome of the hospitalization (discharge or death), were recorded from the electronic medical record. Laboratory results were abstracted from the collection date, or if unavailable the closest to that of research blood collection, mostly within 48h. Clinical and demographic information including age, BMI and whether patients were on immunosuppressant or anti-inflammatory drugs are summarized across the groups and displayed individually, in Dataset S1.

Cell culture

Cells were treated for 24h with: CCL2 (60 ng/mL, BioLegend, cat#571402); CCL8 (20 ng/mL, BioLegend, cat#581602); CCL20 (10 ng/mL, BioLegend, cat#583804); CXCL1 (10 ng/mL, BioLegend, cat#574402); CXCL2 (5 µg/mL, BioLegend, cat#582002); CXCL10 (1 µg/mL, BioLegend, cat#573502); CXCL11 (20 ng/mL, BioLegend, cat#574902); galectin 9 (150 ng/mL, BioLegend, cat#557302); granzyme B (3 µg/mL, BioLegend, cat#554902); IFNγ (50 ng/mL, BioLegend, cat#570202); IFNλ (50 ng/mL, BioLegend, cat#711204); IL1β (20 ng/mL, BioLegend,

cat#579402); IL1RL (250 ng/mL, BioLegend, cat#557904); IL6 (20 ng/mL, BioLegend, cat#570802); IL8 (50 ng/mL, BioLegend, cat#574208); IL18 (50 ng/mL, BioLegend, cat#592102); IL32α (0.1ng/mL, BioLegend, cat#551002); TNFα (25 ng/mL, BioLegend, cat#570102), PLA2G7 (10 ng/mL, BioLegend, cat#762902); Serpin E1 (2.5 μg/mL, BioLegend, cat#753802); L-arginine (2mM, Sigma #A5006); L-asparagine (2mM, Sigma #A4159); D2-HG (20 mM, Cayman chemicals #11605); L-Kynurenine (100 μM, Sigma #K8625); Sodium L-lactate (20 mM, Sigma #71718); nicotinamide (20 mM, Sigma #N0636). Cells cultured under hypoxia were placed in a hypoxic chamber (5% O_2) for 24h.

Flow cytometry

Cells were stained using the following cell surface antibodies: CD14 Pacific Blue (clone M5E2, BioLegend cat# 301815, 2:100 dilution); CD19 Pacific Blue (clone H1B19, BioLegend cat#302224, 2:100 dilution); CD3 AF700 (clone OKT3, BioLegend, cat# 317340, 2:100 dilution); CD4 PerCP-Cy5.5 (clone OKT4, BioLegend, cat# 317428, 2:100 dilution); CD127 AF488 (clone A019D5, BioLegend, cat# 351314, dilution 3:100); CD25 PE-Cy7 (clone BC96, BioLegend, cat# 302611, dilution 3:100); KLRG1 APC-Cy7 (clone 2FI/KLRG1, BioLegend, cat# 138426, 2:100 dilution); CD279 (PD-1) BV650 (clone EH12.2H7, BioLegend, cat# 329950, dilution 2:100); CD45RA BV510 (clone HI100, BioLegend, cat# 304142, dilution 2:100). The following intracellular antibodies were also used: FoxP3 APC (clone PCH101, Invitrogen, cat# 17-4776-42, dilution 4:100); Tbet BV605 (clone 4B10, BioLegend, cat# 644817, dilution 4:100); Bcl6 PE/Dazzle (clone 7D1, BioLegend, cat# 358510, dilution 4:100).

RNA-seq

Low-input RNAseq: Smart-seq2 libraries were prepared as previously described (1) with slight modifications. Briefly, total RNA was captured and purified on RNAClean XP beads

(Beckman Coulter). Polyadenylated mRNA was then selected using an anchored oligo(dT) primer (50 –AAGCAGTGGTATCAACGCAGAGTACT30VN-30) and converted to cDNA via reverse transcription. First strand cDNA was subjected to limited PCR amplification followed by Tn5 transposon-based fragmentation using the Nextera XT DNA Library Preparation Kit (Illumina). Samples were then PCR amplified for 12 cycles using barcoded primers such that each sample carries a specific combination of eight base Illumina P5 and P7 barcodes for subsequent pooling and sequencing. Paired-end sequencing was performed on an Illumina NextSeq 500 using 2 x 38bp reads with no further trimming. Reads were aligned to the human genome (GENCODE GRCh38 primary assembly and gene annotations v27) with STAR 2.5.4a (https://github.com/alexdobin/STAR/releases). The ribosomal RNA gene annotations were removed from GTF (General Transfer Format) file. The gene-level quantification was calculated by featureCounts (http://subread.sourceforge.net/). Raw read counts tables were normalized by median of ratios method with DESeq2 package from Bioconductor (https://bioconductor.org/packages/release/bioc/html/DESeq2.html) and then converted to GCT and CLS format.

Quality control. Samples with less than 1 million uniquely mapped reads were automatically excluded from normalization to mitigate the effect of poor-quality samples on normalized counts. Samples having fewer than 8,000 genes with over ten reads were also removed from the data. We screened for contamination by using known cell type specific transcripts (per ImmGen ULI RNAseq and microarray data). Finally, the RNA integrity for all samples were measured by median TIN across human housekeeping genes with RSeQC software (http://rseqc.sourceforge.net/#tin-py). Samples with TIN < 45 were removed from the data set prior to downstream analysis.

Batch correction. In order to analyze simultaneously the two RNAseq batches, the read counts tables from these two batches were first combined for all the Treg samples and for all the

Tconv samples separately. Then, the batch effects were corrected by using Combat method from sva package.

Viral reads mapping: The SARS-Cov-2 genome sequence and annotation were obtained from NCBI (https://www.ncbi.nlm.nih.gov/sars-cov-2/). Reads were aligned by STAR 2.5.4a with the parameters suggested in Kim et al.(2). The gene-level quantification was calculated by featureCounts (Subread 1.6.2). Read counts tables were normalized by median of ratios method with DESeq2 package (3)

Regression Linear model. Related to the epidemiology of the disease, there was a strong sex-bias in severe patients in our dataset (predominantly male). Thus, we performed a linear regression (*glm()* function in R) using log-transformed expression as the response variable, and severity and sex as explanatory variables. We removed 45 genes highly correlated with sex, which were no longer associated with severity once adjusted.

Differential gene expression. After quality control and regression, genes with a minimum reads count of 20 in more than 20% of samples from a population (Treg or Tconv) were retained. We used an uncorrected t-test to compute differential gene expression between the different groups from the normalized read counts dataset. Genes with a FoldChange >2 or <0.5 and p-value < 0.05 were selected for further analysis.

Computation of signatures and module indexes: the different COVID-19 (SCTS), Treg signature, TITR, and modules indexes were calculated for each donor by averaging the normalized expression (versus mean of all HD) of all genes belonging to each signature.

Geneset enrichment analysis. CD4+ Tcell gene signatures were curated from published and relevant datasets, as described (4). Only datasets containing replicates were used. To reduce noise, genes with a coefficient of variation between biological replicates <0.6-0.8 in either comparison groups were selected. Up- and downregulated transcripts were defined as having a fold change in gene expression >1.5 or <0.6 and a t-test p-value <0.05, limited to the top 300

genes by signature. Other signatures were obtained from extracting all CD4+ T cells signatures from the MSigDB C7 Immunologic collection (5). Geneset enrichment analysis with the COVID-19 signature was performed using hypergeometric distribution and type I error was controlled using FDR. Signatures with FDR <10% and an overlap with COVID-19 signature >10 genes were considered as significant and are reported in table S3.

SUPPLEMENTARY REFERENCES

1. S. Picelli, et al., Full-length RNA-seq from single cells using Smart-seq2. *Nat Protoc.* **9,** 171-181 (2014).
2. D. Kim, et al., The architecture of SARS-CoV-2 transcriptome. *Cell* **181,** 914-921 (2020).
3. M. I. Love, W. Huber, S. Anders, Moderated estimation of fold change and dispersion for RNA-seq data with DESeq2. *Genome Biol.* **15,** 550 (2014).
4. D. Zemmour, et al., Single-cell gene expression reveals a landscape of regulatory T cell phenotypes shaped by the TCR. *Nat Immunol* **19,** 291-301 (2018).
5. J. Godec, et al., Compendium of Immune Signatures Identifies Conserved and Species-Specific Biology in Response to Inflammation. *Immunity* **44,** 194-206 (2016).
6. A. Ferraro, et al., Interindividual variation in human T regulatory cells. *Proc Natl Acad Sci U S A* **111,** E1111-E1120 (2014).
7. A. M. Magnuson, et al., Identification and validation of a tumor-infiltrating Treg transcriptional signature conserved across species and tumor types. *Proc Natl Acad Sci U S A* **115,** E10672-E10681 (2018).
8. J. R. Dispirito, et al., Molecular diversification of regulatory T cells in nonlymphoid tissues. *Sci Immunol* **3,** eaat5861 (2018).

Supplementary figure 1

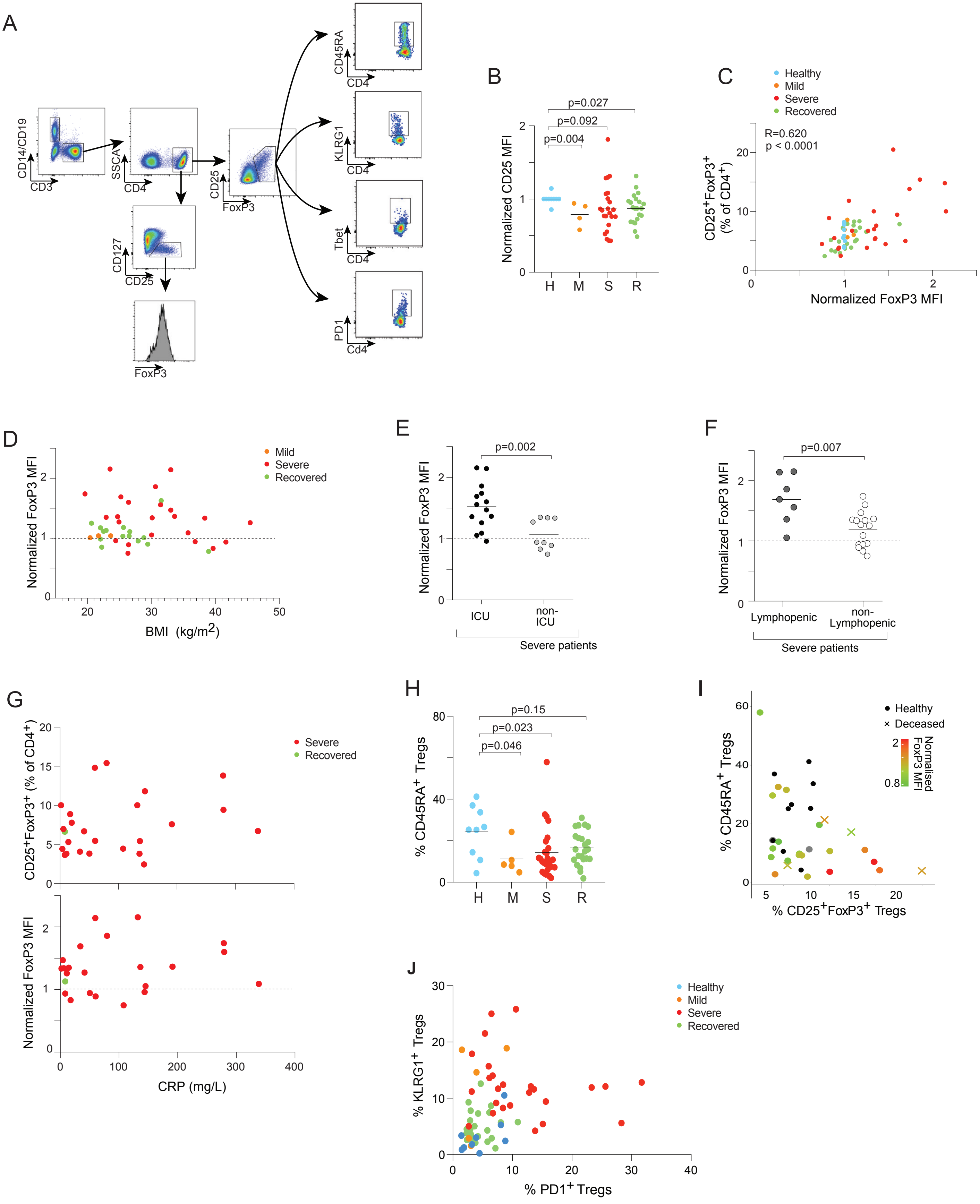

Fig. S1. **Flow cytometry phenotypes of Tregs from severe COVID19 patients**

(A) Representative flow cytometry plots of the gating strategy for Tregs, FoxP3 MFI and Treg markers. (B) Expression of CD25, measured as normalized MFI in $CD127^{lo}$ $CD25^{+}$ Tregs across the whole cohort. (C) Correlation between percentage of $CD25^{+}$ $FoxP3^{+}$ Tregs and FoxP3 expression as MFI across the whole cohort. (D) Correlation between FoxP3 expression as MFI in Tregs and BMI across the whole cohort. (E,F) Expression of FoxP3 as MFI in Tregs from severe COVID-19 patients, comparing intensive care unit (ICU) admission and lymphopenia, determined as a B cell or T cell percentage significantly lower than the average. (G) Correlation between CRP level in COVID patients and percentage of Tregs (top) or expression of FoxP3 as MFI (bottom). (H) Proportion of CD45RA+ Tregs as determined by flow cytometry across the whole cohort; p-values from Mann–Whitney test. (I) Correlation between percentage of $CD25^{+}FoxP3^{+}$ Tregs (x-axis), percentage of CD45RA+ Tregs (y-axis) and FoxP3 expression as MFI (color gradient) within severe COVID-19 patients. Healthy controls depicted in black dots and patients with fatal outcome by a cross. (J) Correlation between KLRG1+ Tregs and PD1+ Tregs as determined by flow cytometry, across the whole cohort. All P-values were computed from Mann–Whitney test.

Supplementary figure 2

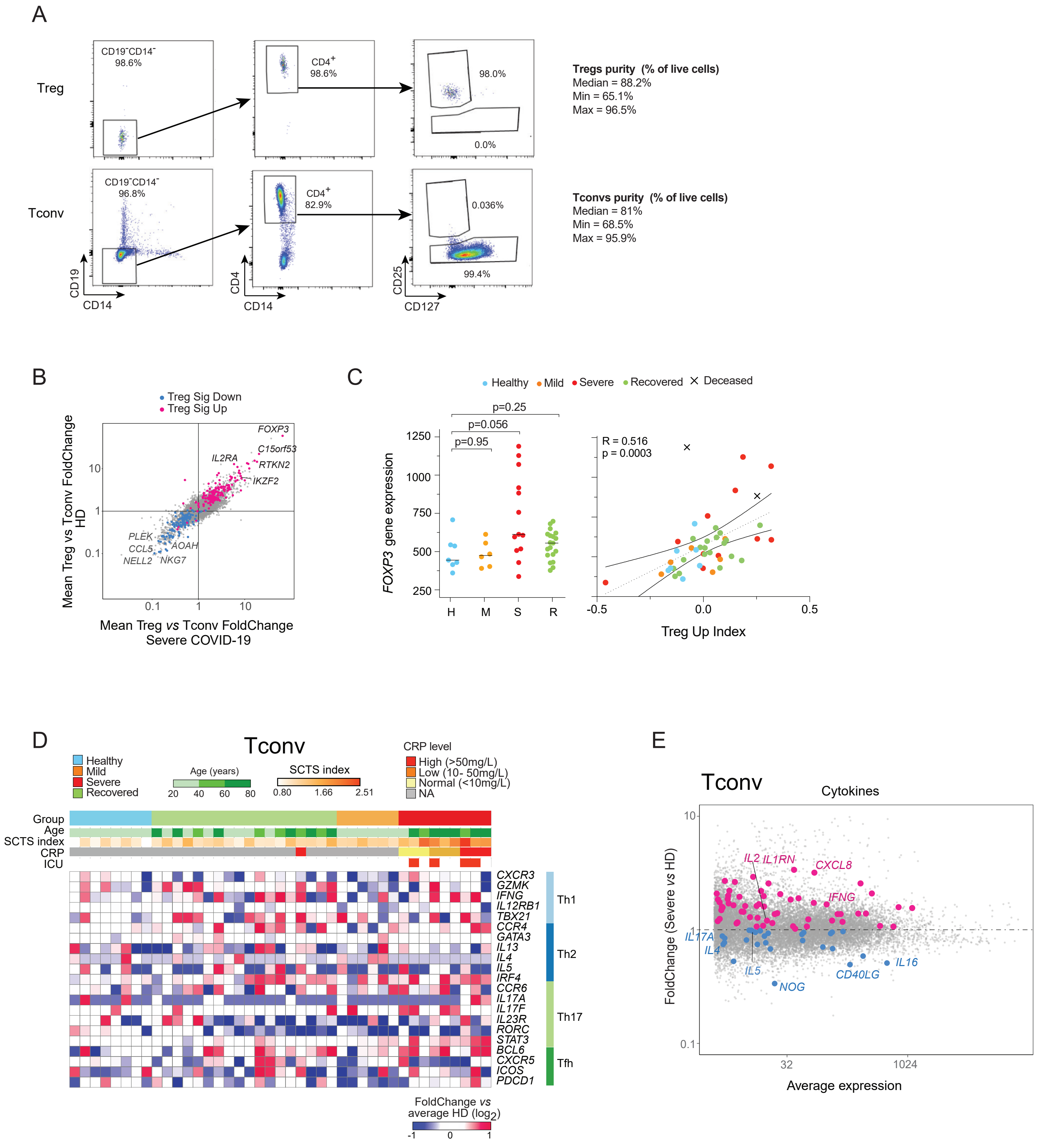

Fig. S2. **Transcriptomic profile of CD4+ Treg and Tconv cells**

(A) Representative flow cytometry plots showing a sample of the final isolated fractions of magnetically-purified purified Tregs (top) and Tconvs (bottom). The median and min/max of purity for each isolated population in the whole cohort is listed. (B) Comparison between the Treg/Tconv FoldChange in HD (y-axis) versus severe COVID-19 (x-axis); Treg signature genes (6) are highlighted. (C) Gene expression of *FOXP3* in Tregs across the whole cohort (left) and its correlation with the TregUP index (right). Patients with fatal outcome depicted by a cross. R statistic from a Pearson correlation procedure and p-values from Mann–Whitney test. (D) Expression heatmap of the canonical T helper genes (Th1, Th2, Th17 and T follicular helper) in Tconvs from each patient versus average expression in HD. One column per patient, with severity groups color-coded, and with color-gradients for age, *Severe COVID19 Treg Signature* (SCTS) index and systemic level of CRP. (E) FoldChange vs average expression (MA) plot from severe COVID-19 patients' Tconvs compared to HD. Upregulated (pink) and downregulated (blue) cytokine transcripts highlighted.

Supplementary figure 3

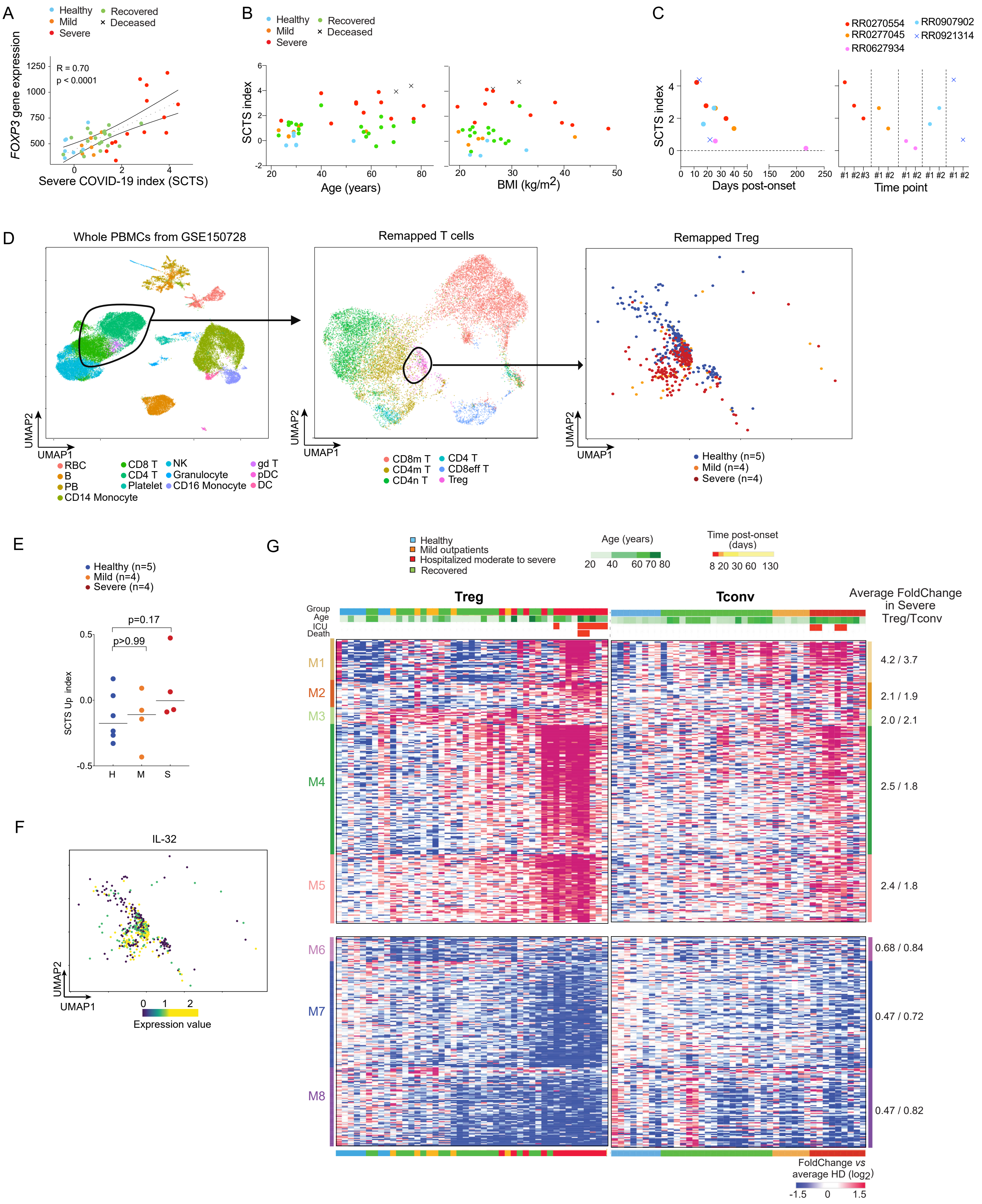

Fig. S3. **Elements of the severe COVID-19 Treg signature**

(A) Correlation between *FOXP3* gene expression and the *Severe COVID19 Treg Signature* (SCTS) index across the whole cohort. (B) Correlation between SCTS index and age (left) or BMI (right) across the whole cohort. Patients with fatal outcome depicted by a cross. R statistic and p-value from a Pearson correlation procedure. (C) Correlation between SCTS index and days post disease onset (left). Right panel depicts the SCTS index for sequential samples acquired from each patient (D) Extraction strategy of the Tregs population from scRNAseq dataset (GSE150728). COVID-19 patients' PBMCs were displayed as a 2D UMAP at different levels of extraction: the whole PBMCs (left panel), T cells (middle panel) and Tregs (right panel). Samples are color-coded by cell type, except for Tregs which are color-coded by patient group. (E) Quantification of the SCTS index for each patient among the different groupsp-values from Mann–Whitney test. (F) Same 2D UMAP than in A (right panel) but indicating *IL32* expression among the COVID-19 Tregs dataset. (G) Heatmap of the differentially expressed genes identified in Fig 3B ($p<0.05$ (t-test), FC >2 or <0.5) across all groups in Tregs (left) versus Tconvs (right). Each column represents one sample. Top ribbons indicate for each individual: severity group, age, ICU admission and final outcome (deceased, in red). Left ribbon indicates the different modules and the right ribbon the average FoldChange in severe Tregs versus severe Tconvs.

Supplementary figure 4

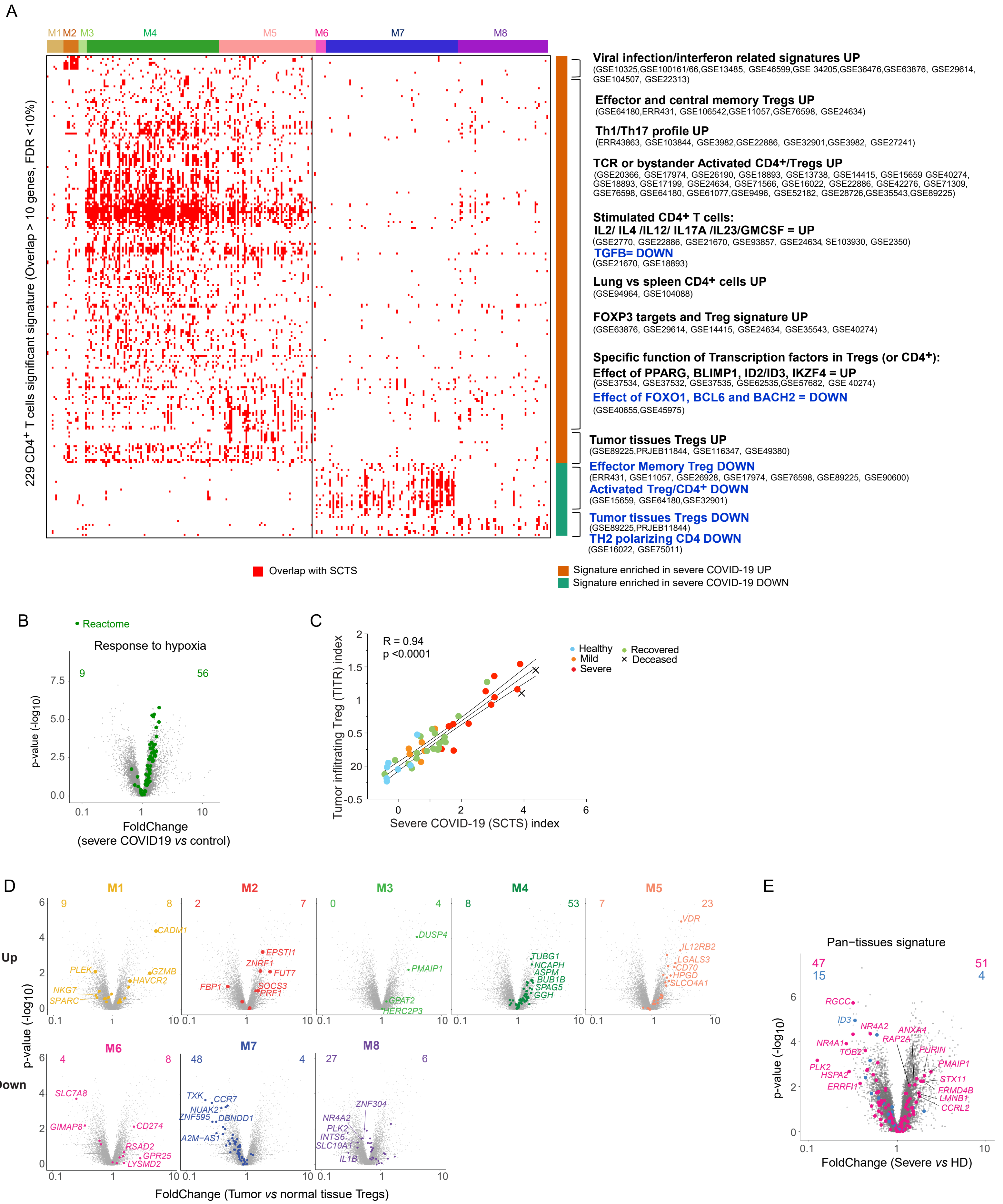

Fig. S4. **Similarities between tumor and severe COVID-19 Treg**

(A) Heatmap of the overlap between the *Severe COVID19 Treg Signature* (SCTS) (defined in Fig3A), and other relevant immunological signatures (hypergeometric test FDR <10% and more than 10 genes in overlap). The top ribbon indicates the modules (defined in Fig3C). Annotation of common clusters of signatures and the signatures belonging to these clusters with their GEO dataset ID at the right. All signatures and the overlap parameters can be found in Table S3. (B) FoldChange vs p value (volcano) plot of normalized expression in Tregs from severe COVID-19 patients compared to HD. Genes from the "*Cellular response to Hypoxia"* signature from the Reactome database are highlighted. (C) Correlation between the TITR (Tumors infiltrating Tregs index) and the SCTS index. Patients with fatal outcome depicted by a cross. R statistic and p-value from Pearson correlation procedure. (D) FoldChange vs p value (volcano) plots of normalized expression in Tregs from colorectal cancer (CRC) vs normal colon Tregs (7). SCTS modules from Fig 3C are highlighted and the numbers of their genes up and down are annotated. (E) FoldChange vs p value (volcano) plot of normalized expression in Tregs from severe COVID-19 patients compared to HD. Signature genes from Pan Tissues Tregs (8) are highlighted.

Supplementary figure 5

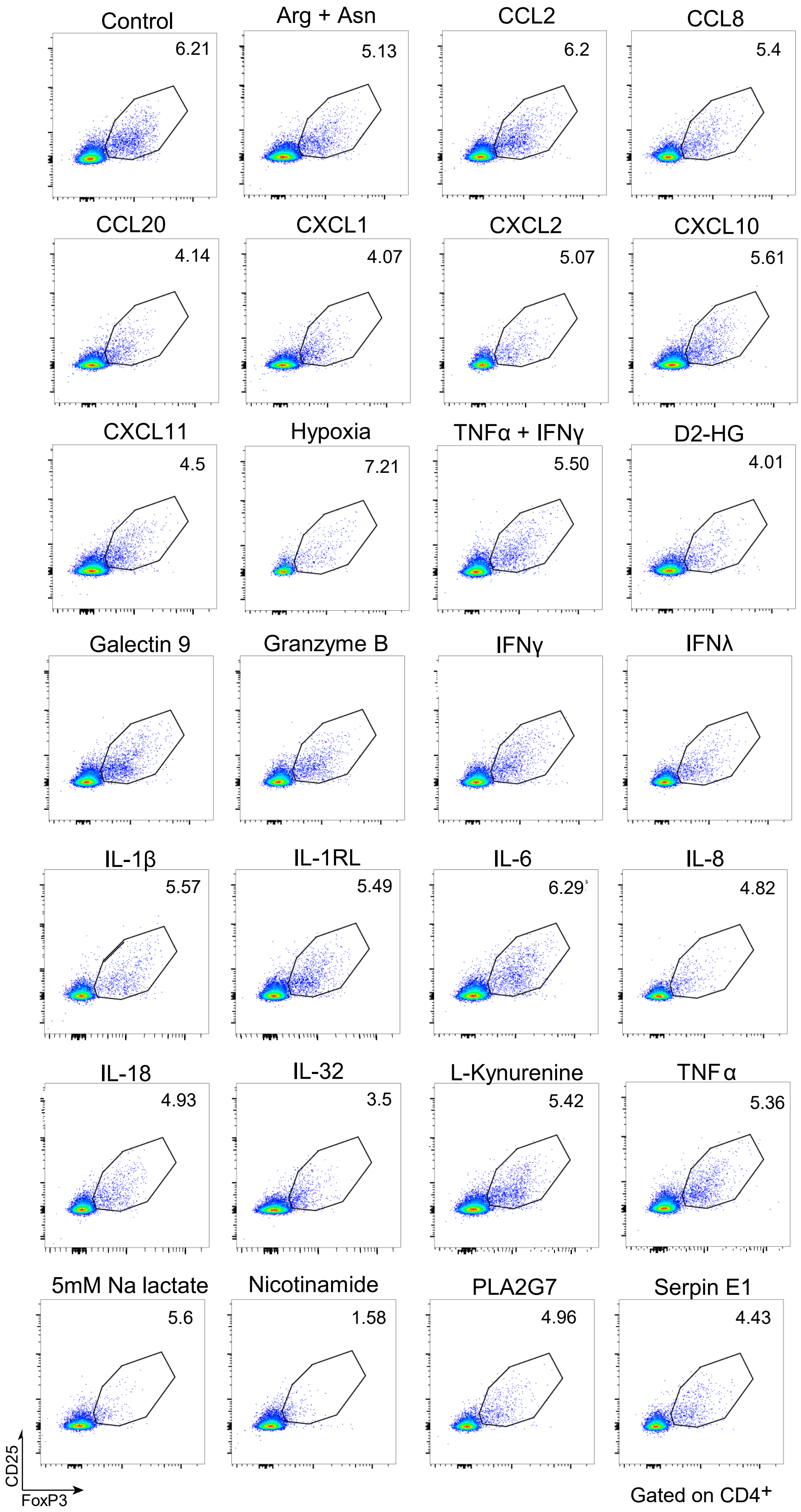

Fig. S5. **Flow cytometry profiles of Tregs following cytokine/chemokine/metabolite stimulation.**

PBMCs from HDs were left untreated (ctrl) or treated for 24h with the listed cytokine, chemokine or other mediator, or cultured in a hypoxic chamber. Treg percentage and FoxP3 expression was assessed by flow cytometry. Representative of two experiments.

SI DATASET LEGENDS

Dataset S1. Clinical, biological and analytic characteristics of the cohort (n=68)

(A) Clinical characteristics of COVID-19 patients and healthy donors (summary, n=68) (B) Individual clinical and biological data (n=68) - at collection date (C) Samples included in the Flow cytometry dataset (n=63): key parameters (D) Samples included in the Low input RNAseq dataset: purity and quality (Tregs n=45 & Tconvs n=41) (E) Viral reads in the Treg/Tconv RNAseq dataset. NA: Not available.

Dataset S2. *Severe COVID19 Treg Signature* (SCTS) genes with their average Severe/HD fold change in Tregs

Dataset S3. CD4+ signatures significantly enriched in the *Severe COVID19 Treg Signature* (hypergeometric test FDR <10% and more than 10 genes in overlap).

MGH COVID-19 Collection & Processing Team participants

Collection Team
Kendall Lavin-Parsons
Blair Parry
Brendan Lilley
Carl Lodenstein
Brenna McKaig
Nicole Charland
Hargun Khanna
Justin Margolin

Processing Team
Anna Gonye
Irena Gushterova
Tom Lasalle
Nihaarika Sharma
Brian C. Russo
Maricarmen Rojas-Lopez
Moshe Sade-Feldman
Kasidet Manakongtreecheep
Jessica Tantivit
Molly Fisher Thomas
